# Sparse Firing and Connectivity in Neurogenesis Improve Pattern Separation and Memory in a Simulated Dentate Gyrus

**DOI:** 10.1002/hipo.70146

**Published:** 2026-11-06

**Authors:** Daniel A. C. van der Meer, Raoul P. P. P. Grasman

**Affiliations:** ^1^ Department of Psychology University of Amsterdam Amsterdam the Netherlands

**Keywords:** adult neurogenesis, dentate gyrus, memory interference, pattern separation, restricted Boltzmann machine, spiking neural network

## Abstract

Adult neurogenesis, the birth of new neurons continuing into adulthood, is believed to play a role in pattern separation during the encoding of episodic memories, particularly in the dentate gyrus of the hippocampus. However, computational models of the dentate gyrus that incorporate neurogenesis have shown inconsistent results and often omit learning and memory processes. We incorporated the dynamics of aging dentate granule cells into a spiking restricted Boltzmann machine model of the dentate gyrus to study the effects of neurogenesis on pattern separation and memory interference. Our simulations show that including neurogenesis and modeling the detailed changes over the granule cell life cycle leads to a general improvement in pattern separation. At the same time, both proactive and retroactive interference are greatly reduced. These results support the view that sparse firing in mature granule cells and sparse connectivity in young granule cells are critical factors in establishing a functional role for neurogenesis in the dentate gyrus.

## 1 Introduction

Pattern separation (and conversely pattern completion) is thought to be fundamental to mammalian memory (Chawla et al. 2005; O'Reilly and McClelland 1994; Rolls 1987). This process of distributing information efficiently within an available encoding space allows for less overlap between conceptually different memories while removing unnecessary redundancy for similar ones. This should, at least in theory, greatly facilitate the storage and retrieval of new and old memories. One of the mechanisms by which effective pattern separation could achieve this is via the reduction of memory interference (Kesner 2013). Intuitively, increased pattern separation (and thus decreased overlap between memory traces) decreases the likelihood of overwriting old memories (retroactive interference) as well as the likelihood of old memories interfering with the formation of new memories (proactive interference).

The formation of memory has been extensively linked to the archicortex, and more specifically to the hippocampal formation, for several decades (Knierim and Neunuebel 2016; Marr 1971; Neunuebel and Knierim 2014; Wills et al. 2005). In a seminal theoretical framework, Marr (1971) advanced the field by proposing a computational model of memory storage within the archicortex, delineating the necessary neurobiological constraints for effective memory encoding and retrieval (Marr 1971; Willshaw and Buckingham 1990). Central to this model are principles such as sparse synaptic connectivity, Hebbian synaptic plasticity, and the formation of auto‐associative networks within the hippocampus, particularly within area CA3, which together enable pattern completion and content‐addressable memory.

The dentate gyrus (DG) is the first processing stage of the hippocampus, with signals mostly coming from the entorhinal cortex (EC; Amaral et al. 2007). The DG itself projects towards the CA3 (via mossy fibers), which projects onwards to CA1 regions of the hippocampus, which in turn project back to the EC, completing the trisynaptic circuit. As an area with relatively few cell types, although having more cells in total than the EC (Amaral et al. 1990, 2007), the DG has become an area of interest regarding the possibility of serving as a pattern separation stage in encoding episodic memories (Bakker et al. 2008; O'Reilly and McClelland 1994; Yassa and Stark 2011). This view is supported by the relatively unidirectional chain of connections (Amaral et al. 1990) and the comparably low activity of its neurons (Chawla et al. 2005; Jung and McNaughton 1993), which, together with its relative abundance of cells, serves as a plausible prerequisite for sparse encoding (Becker 2005). The main cell type found in the DG is the dentate granule cell (GC). These receive excitatory input from the EC via the perforant pathways (PP), and project excitatory efferents towards the CA3 region via mossy fibers. The GCs themselves are arranged in lamellar clusters where several GCs receive inhibitory inputs from a single basket cell (BC) and hilar perforant path (HIPP) associated cell (Andersen et al. 1971; Myers and Scharfman 2009).

A key factor driving recent research interest in the DG is its association with adult neurogenesis (ANG). Dentate granule cells (GCs), which constitute the main mass of the DG, have been shown to be continuously replaced throughout life in rats, as well as in humans (Besnard and Sahay 2015; Boldrini et al. 2018; Denoth‐Lippuner and Jessberger 2021; Drew et al. 2013; Eriksson et al. 1998; Ming & Ming and Song 2005; Moreno‐Jiménez et al. 2019; Spalding et al. 2013) (but also see Sorrells et al. 2018). Although the rate of neurogenesis is decreasing with age in most mammals (Boldrini et al. 2018; Kempermann et al. 1998), it is believed to be important for learning (Kempermann and Gage 2002). Furthermore, some evidence suggests that in humans neurogenesis is relatively stable throughout the life span of healthy adults (Moreno‐Jiménez et al. 2019). Specifically, the change in activation and connectivity profile during the maturation of GCs is thought to be crucial for pattern separation (Dieni et al. 2016; Finnegan and Becker 2015). Immature GCs (imGCs) still have a high membrane resistance and relatively depolarized resting potentials, resulting in strong excitability (Liu et al. 1996; Schmidt‐Hieber et al. 2004; Snyder et al. 2001). At the same time, they are much more sparsely connected than mature GCs (mGCs), meaning they have yet to develop most of their synaptic connections (Piatti et al. 2013).

Various empirical studies on these dynamics have informed theoretical frameworks regarding their relevance for pattern separation and memory formation in biological settings and empirical studies show that rats with reduced neurogenesis show worse performance on the Morris water maze task (Dupret et al. 2008; Snyder et al. 2005; Zhang et al. 2008), as well as impaired fear conditioning (Saxe et al. 2006). On the other hand, at least some of the effects regarding memories could not be found in other studies (Saxe et al. 2007; Shors et al. 2002).

Current theories often assume that neurogenesis plays a role in the encoding of new memories and that in turn, reduced neurogenesis impairs memory formation. There are however some differences in terms of the precise mechanism that is hypothesized to affect the relationship between neurogenesis and memory formation. Some theoretical approaches reason that ANG should protect against proactive interference by facilitating the encoding of new items (Luu et al. 2012), whereas others hypothesize that ANG should prevent retroactive interference in old memories, possibly caused by the formation of new ones (Becker 2005). Experimental results support the latter by showing that rats with suppressed neurogenesis remember less of a learned task when exposed to subsequent interfering stimuli compared to rats without suppressed neurogenesis (Winocur et al. 2012), supporting the idea that ANG should generally prevent memory interference, but empirically establishing a connection to pattern separation is hard. Given that pattern separation causes reduced overlap in memories, it is seen as a plausible mechanism via which neurogenesis could affect memory interference (Leal and Yassa 2018; Marr 1971; Treves and Rolls 1994; Yassa and Stark 2011).

Several computational models have been developed to formally describe neurogenesis dynamics. By simulating the growth of a rat DG over several months, a model proposed by Aimone et al. (2009) suggests a nuanced function of the aging related properties of GCs. While the development of imGCs promoted pattern integration for stimuli over shorter temporal scales, the authors also found an increase in pattern separation over longer time scales. With regards to the specific dynamics of aging GCs, they concluded that the heightened excitability of imGCs actually contributes to a decrease in pattern separation, leading to an overall reduction of pattern separation with the inclusion of imGCs. However, this finding is not easy to integrate with findings of experimental studies that attempt to alter ANG in rodents, as well as to findings from studies in humans (Clelland et al. 2009; Creer et al. 2010; Déry et al. 2013). Aimone et al. (2009) resolve this apparent discrepancy by nuancing between pattern integration and pattern completion, proposing that pattern integration (the limitation of separation of distinct inputs) should be seen as separate from pattern completion (production of equal output for different inputs) which takes place in other downstream areas of the hippocampus. Other simulations that included detailed neuron dynamics were conducted by Faghihi and Moustafa (2016) and by Kim and Lim (2024). While Faghihi and Moustafa (2016) found increased pattern separation for certain rates of neurogenesis, Kim and Lim (2024) did not find any advantage of incorporating dynamics of imGCs in their model when compared to a model with only mGCs. Kim and Lim (2024) found that mGCs might act as pattern separators, while imGCs acted more as pattern integrators through high excitability.

One issue regarding the comparison between simulation studies and empirical findings is that simulation studies mostly rely on measures of pattern separation, whereas empirical studies usually rely on measures of memory interference. While most of these simulation studies incorporate updating of the synaptic connections, they often lack a memory storage of learned representations. This makes it hard to discern the influence of ANG on pattern separation from its influence on memory interference. This distinction could however be very important as pattern separation might not automatically correspond to less memory interference (Finnegan and Becker 2015).

Generally, omitting learning neglects a critical dual role of the hippocampus: not only does it facilitate the encoding of new experiences, but it also serves as a temporary storage site for episodic memories prior to systems‐level consolidation (Burgess et al. 2002; O'Reilly and McClelland 1994; Vargha‐Khadem et al. 1997). Models lacking a learning component make a direct comparison between empirical results and simulation studies difficult to interpret.

To account for that, Finnegan and Becker (2015) and Finnegan et al. (2017) introduced an adapted version of the Restricted Boltzmann Machine (RBM) as a model for the DG to investigate mechanisms of pattern separation in a learning system. RBMs are dynamical systems that consist of a bipartite graph of artificial neurons that store memory items as attractors within their weights. Learning in an RBM takes place by means of auto‐association using a Hebbian learning rule. This makes them a close fit for modeling the putative role of the hippocampus. In their simulation, they included the characteristics of aging GCs by incorporating sparse firing, sparse connectivity, and a dynamically changing learning rate to accommodate the evolving excitability of imGCs and neurogenesis of DG cells. In the two models they put forward, Finnegan and Becker (2015) and Finnegan et al. (2017) respectively found that neurogenesis decreases memory interference in line with the possible benefits of ANG, but unexpectedly, they also found a *decrease in pattern separation*. The authors take their findings to argue for a change of perspective on the necessity of pattern separation on memory interference. However, since pattern separation should be one means of reducing memory interference, and with most theories claiming that neurogenesis should increase pattern separation, their results are somewhat counter‐intuitive.

The simulations in Finnegan and Becker (2015) and Finnegan et al. (2017) extend the earlier computational work mentioned previously, and provide the modeling framework of RBMs as a foundation for a more realistic modeling paradigm that includes (Hebbian) learning. A number of departures from biological reality can be identified in their simulations. In the current paper we seek to address these aspects of their simulation to match biological findings more closely. Specifically, we identify the following unclear and/or implausible choices: First, in Finnegan and Becker (2015), it is not entirely clear how large the percentage of imGCs was in the simulation. Appropriate values for this percentage would range from a few percent of the total GC population at any given time (in adults Piatti et al. 2013; Spalding et al. 2013), to up to 10% (a cumulative proportion of all surviving adult‐born neurons; Kim and Lim 2024). Second, mature granule cells were reportedly fully connected to the EC, which substantially deviates from actual findings regarding the connectivity between EC and DG, as each granule cell is estimated to receive input from only approximately 1%–2% of entorhinal cortex neurons, see Amaral et al. (1990). As a similar shortcoming with respect to biological plausibility, other studies seem to add quite a high number of imGCs throughout a simulation (Aimone et al. 2009), are more than doubling the total number of neurons in their network, which limits the interpretation of the model's results to very large timescale scenarios and elevated neurogenesis rates that are only realistic for very young rats/humans (Kempermann et al. 1998). This in turn might exaggerate the pattern integrating effect of highly excitable imGCs in the overall system (Kim and Lim 2024).

Furthermore, previous models often rely on artificial networks in which the activity is stimulus‐driven (Vogels et al. 2005; Yuste 2015). Without any input, there is no activity and no memory represented. Most activity in the brain is driven internally. A core complexity in biological neural networks pointed out by Vogels et al. (2005) is that this internally generated activity competes with the selectivity to external stimuli. One consequence of internal activation is that networks exhibit attractor dynamics, which allow them to continue generating activity patterns corresponding to a stimulus even long after that stimulus has disappeared (Brader et al. 2007; Vogels et al. 2005; Wang 2003). In a spiking network with continuously operating synaptic plasticity, this same internal activity provides an additional benefit: spontaneous reactivation of learned attractor states between stimulus presentations drives further weight consolidation—a process analogous to hippocampal memory replay (e.g., Nádasdy et al. 1999), that is entirely absent in stimulus‐driven models. None of the studies discussed so far have considered biophysically detailed spiking neuro‐dynamics with learning or auto‐associative retrieval.

Within the RBM framework, more recently, adaptations have been proposed that allow for biophysically realistic spiking neurons in RBMs (so‐called spiking RBM or sRBM, Hinton and Brown 1999; Neftci et al. 2014). These adaptations entail higher realism in terms of biophysical neuronal activity and synaptic plasticity dynamics. Our goal here is therefore to revisit the findings of Finnegan and Becker (2015) using such spiking RBMs.

By using a spiking neural network that implements spike‐timing‐dependent plasticity (STDP) to update synaptic weights, we aim to develop a more neurologically plausible model of the DG and to elucidate the discrepancies observed in earlier studies. In particular, we model the age‐dependent characteristics of maturing GCs more realistically by explicitly incorporating membrane potential and spiking dynamics of individual neurons. Further, we model dynamics on a shorter timescale than some previous studies. ANG is a process taking place throughout life, with its major change happening during early ages of development. Here, we limit neurogenesis rates to levels that can be plausibly expected in relatively short time spans of several weeks instead of months. Additionally, by measuring pattern separation and memory interference, we intend to facilitate the comparison between empirical work and our simulation results. In doing so, our model is the first to embed biophysically grounded spiking dynamics within an auto‐associative attractor memory framework, enabling joint and direct measurement of both pattern separation and memory interference in the same dynamical system. Contrary to earlier studies and in line with empirical results, we expect a negative relationship between pattern separation degree and memory interference in the sense that the former should prevent the latter.

## 2 Methods

This model builds on the architecture of Finnegan and Becker (2015) and Finnegan et al. (2017), replacing stimulus‐driven neurons with biophysically plausible spiking neurons as described in Neftci et al. (2014).

### 2.1 Spiking Restricted Boltzmann Machines

Restricted Boltzmann machines (RBM) are energy‐based network models that can be trained to represent a probability distribution of an input. RBMs consist of a so‐called visible layer of nodes (which in Finnegan et al. 2017 represent afferent inputs coming from the EC) and a hidden layer of nodes (which in Finnegan et al. 2017 represent GCs in the DG). Usually, each visible node is connected to each hidden node with a weight that determines the impact an activation of the visible neuron has on the hidden one and vice versa. The contrastive divergence (CD) algorithm proposed by Hinton (2002) exploits the bipartite graph structure by using rounds of Gibbs sampling to efficiently sample the state of the hidden units given an input pattern. These are necessary for Hebbian updating of the connection weights between the visible and hidden units. This allows for efficient storage of input stimuli in the form of a representation in the weights between visible and hidden units. Activation of the hidden layer enables reconstruction and pattern completion given a limited input stimulus.

RBMs are interesting as models for biological neural networks due to the Hebbian learning characteristics their training exhibits as well as their ability to learn stimulus representations in an unsupervised as well as supervised manner. Importantly, RBMs are dynamical systems that store memory items as attractor states, a key characteristic of the hippocampal dynamics (Rolls 2007).

Several approaches have been proposed to construct spiking RBMs (Hinton and Brown 1999; Neftci et al. 2014; Neil 2013; O'Connor et al. 2013). These models differ from classic RBMs by incorporating time and the elicitation of spikes (i.e., action potentials). We use the approach of Neftci et al. (2014) as they provide high‐quality and well‐documented software. Their implementation uses leaky integrate‐and‐fire (LIF) neurons that are connected via connecting synapses. These LIF neurons represent a potential for each of the visible and hidden nodes which is in its dynamics comparable to the membrane potential in real neuron cells. The potential of the LIF neurons is governed by the following (stochastic) differential equation:

(1) $${\mathrm{Cdu}}_{i}=-g_{L}u_{i}\mathrm{dt}+I_{i}\left(t\right)\mathrm{dt}+\mathrm{\sigma d\xi}\left(t\right),\;u_{i}\left(t\right)<\theta ,$$

where the change in membrane potential u at time‐point t for neuron i is determined by the membrane capacitance C, a leakage conductance $g_{L}$, an input current $I_{i}$ and a random noise process ξ modeled as Brownian motion. Here we follow Neftci et al. (2014, note that they put the resting potential at 0). The dynamics of these equations allow the potential to rise with each input stimulus (in this case an incoming spike), while a steady leak current (determined by the leak conductance $g_{L}$) leads to a decrease in potential over time. Additionally, the firing threshold θ determines at which point the neuron causes the firing of a spike which is registered in the synapses of the connected neurons. After each spike, the membrane potential is reset to a resting potential $u_{\text{rest}}$.

The input current is modeled as the sum of the currents induced by the stimulus and by the hidden layer, the latter of which is induced by the incoming spikes $h_{j}\left(t\right)$ from the $N_{h}$ neurons in the hidden layer and spontaneous self excitation in the form of Poisson point processes $b_{i}\left(t\right)$:

(2) $$I_{i}\left(t\right)=I_{i}^{d}\left(t\right)+I_{i}^{v}\left(t\right)$$

(3) $$\tau_{\mathrm{syn}}\frac{{\mathrm{dI}}_{i}^{v}}{\mathrm{dt}}=-I_{i}^{v}\left(t\right)+\sum_{j=1}^{N_{h}}q_{h_{\mathrm{ij}}}h_{j}\left(t\right)+q_{b_{i}}b_{v_{i}}\left(t\right).$$

Here, the *q*'s are the synaptic weights, and $\tau_{\mathrm{syn}}$ is the synaptic time constant. Analogous equations hold for the input currents into the hidden layer neurons with synaptic weights $q_{v_{\mathrm{ij}}}$ that result from spike trains $v_{j}\left(t\right)$ of $N_{v}$ neurons in the visible layer (see Neftci et al. 2014 for details).

Learning takes place in the connecting synapses. These synapses transfer signals in form of incoming spikes to the postsynaptic neuron by inducing a postsynaptic potential with its magnitude determined by the synaptic connection strengths $q_{h_{\mathrm{ij}}},q_{b_{i}}$ and $q_{v_{\mathrm{ij}}},q_{b_{j}}$. To allow for online learning in continuous time, Neftci et al. (2014) propose the following equation as a learning rule, which they call event‐driven CD:

(4) $$\frac{d}{\mathrm{dt}}q_{\mathrm{ij}}=A\cdot g\left(t\right)\;{\text{STDP}}_{\mathrm{ij}}\left(v_{i}\left(t\right),h_{j}\left(t\right)\right).$$

Here, the change of the synaptic connection strength $q_{\mathrm{ij}}$ between visible node $v_{i}$ and hidden node $h_{j}$ at a given time‐point t is determined by the pairwise spike‐time dependent plasticity STDP of the given synapse. The modulating factor $g\left(t\right)$ oscillates between −1 and 1 in between a burn‐in period of the length of multiple refractory periods of the neuron during which it is equal to 0. It determines the direction of the weight change, and emulates long‐term potentiation and long‐term depression, respectively (Neftci et al. 2014). STDP determines the plasticity of the synapse as a function of recent postsynaptic activity and incidental spikes from the presynaptic neuron:

(5) $${\text{STDP}}_{\mathrm{ij}}\left(v_{i}\left(t\right),h_{j}\left(t\right)\right)=v_{i}\left(t\right)\left(K*h_{j}\right)\left(t\right)+h_{j}\left(t\right)\left(K*v_{i}\right)\left(t\right)$$

Here, $\left(K*f\right)\left(t\right)=\int_{-\infty }^{t}K\left(t-s\right)f\left(s\right)\mathrm{ds}$, and $K\left(t\right)=\exp\left(-|t/\tau_{\text{STDP}}|\right)$ is a time‐symmetric window function. The (causal) convolutions can be seen as a measure of activation level of the postsynaptic neuron, which takes effect only when spikes arrive from the presynaptic neuron. The factor A>0 serves as a learning rate. In practice, this means that the synaptic connection strength increases if the presynaptic neuron fires shortly before the postsynaptic one while the connection strength is reduced if firing happens in reverse order (see Figure 1). Neftci et al. (2014) show that this event‐driven CD learning rule on average mimics the regular CD learning rule for RBMs. The same rule is also used for adjusting the bias weights $q_{h_{i}}$ and $q_{v_{j}}$. In our implementations all parameters were taken from Neftci et al. (2014) and can be found in Table 1.

**FIGURE 1:**
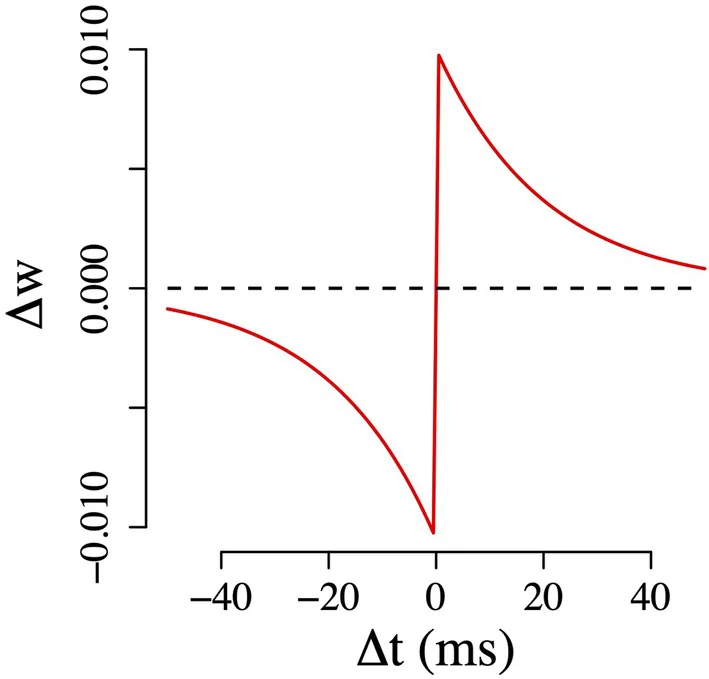
Change of synaptic connection strength as a function of presynaptic firing time. Depending on the order with which two connected neurons are firing, as well as the time between the two events, the synaptic weight either becomes stronger (long term potentiation) or weaker (long term depression). If the first neuron fires shortly before the second one, the weight increases and vice versa. STDP, spike timing‐dependent plasticity.

**TABLE 1:** Base neuron parameters used for all simulations.

Parameter	Description	Value
$\tau_{r}$	Refractory period	4ms
$\nu_{\text{bias}}$	Bias input rate	1000Hz
σ	Noise amplitude	$1\cdot 10^{-9}A/s^{0.5}$
$g_{l}$	Leak conductance	1nS
$u_{\text{rest}}$	Resting potential	0V
C	Membrane capacitance	$1\cdot 10^{-12}\;F$
θ	Firing threshold	100mV

### 2.2 Aging

Following Finnegan et al. (2017), we focus on the characteristics of at least 4 weeks old imGCs, as younger cells are not yet developed enough to produce any action potentials (Zhao et al. 2006). Throughout the simulation, these cells aged until they were mature. As in Finnegan and Becker (2015), all aging neural characteristics were dependent on the cell's maturity (ψ), which followed a Gompertz curve

(6) $$f\left(t\right)=\exp\left(-e^{-\mathrm{ct}}\right).$$

This ranges from zero (*new‐born cell*) to 1 (*mature cell*). The maturity of the cell was then used to scale the aging characteristics within their possible range as the cells matured. Since imGCs only exhibit their specific characteristics for a few weeks (Piatti et al. 2013) we set the steepness parameter c such that their maturation spans roughly 10% of the whole simulation duration. After that, granule cells were mature and exhibited full mGCs characteristics.

#### 2.2.1 Sparsity

As maturing GCs are becoming increasingly connected (Piatti et al. 2013), they are being more and more affected by feedback inhibition (Chawla et al. 2005; Jung and McNaughton 1993; Myers and Scharfman 2009) and their firing becomes more sparse and specialized. A consequence of the latter is sparse coding of input across mature GCs, which has been observed in the DG. Immature GCs, on the other hand, are more broadly tuned while having fewer connections. Similar to Finnegan and Becker (2015) and Temprana et al. (2015), we incorporated these effects of sparse connectivity in imGCs as well as sparse firing in mGCs.

Sparse firing was implemented by means of a sparsity constraint as part of the event‐driven CD learning rule. Specifically, we adjust the event‐driven CD learning rule to include a penalty term that promotes sparsity analogous to the one used in Finnegan and Becker (2015) and Nair and Hinton (2009):

(7) $$\frac{d}{\mathrm{dt}}q_{\mathrm{ij}}=A\cdot g\left(t\right)\text{STDP}\left(v_{i}\left(t\right),h_{j}\left(t\right)\right)-\frac{1}{2}\lambda_{j}\left(t\right)\left[1-g\left(t\right)\right]\left(a_{m}-a_{\text{target}}\right)$$

In Finnegan and Becker (2015) and Nair and Hinton (2009), $a_{m}$ is the probability of the neuron being active, and $a_{\text{target}}$ is an a priori set target probability. The parameter λ>0 is a penalty weight. To get a somewhat biologically plausible way to estimate the average firing activity we use $\left(K*h_{j}\right)\left(t\right)$. However, for stability, instead we obtained an estimate of the proportion of activity neurons in the hidden layer. To do so, we included one leaky helper neuron which integrated all outgoing spikes of the hidden layer over time. Its momentary membrane potential served as a measure for the current average activation $a_{m}$ for all GCs (hidden layer). The target activation level $a_{\text{target}}$ was manually chosen to be smaller than the average activation level $a_{m}$ (see Tables 2 and 3). The sparsity cost $\lambda_{j}\left(t\right)$ was proportional to the age factor $f\left(t\right)$ of the hidden neuron (i.e., it is essentially zero for newborn neurons and fully present for mature neurons). This age‐dependent profile has a plausible biological counterpart in hilar interneurons, particularly HIPP cells, which provide broad feedback inhibition across the GC population and whose coupling to newly born GCs is transiently absent during a critical developmental period (Myers and Scharfman 2009; Temprana et al. 2015).

**TABLE 2:** Parameters for sRBM setup.

Parameter	Value
EC neurons	100
DG neurons	500
DG → EC max connectivity	5%
DG → EC min connectivity	1%
Sparsity cost, λ	0.15
Target activation, $a_{t}$	$1\cdot 10^{-10}$
Percent neurogenesis	15%
Gompertz function growth rate	1
α	0.25
β	0.6
γ	0.15

**TABLE 3:** Implementations of maturing parameters.

Parameter	Implementation
Leak aging factor	$0.5^{*}\left(-\psi \right)+1.5$
Sparsity constraint	$\left\{\begin{array}{ll} -a_{m}\lambda \psi & \text{if}\;g=1\\ +a_{t}\lambda \psi & \text{if}\;g=-1 \end{array}\right.$
Apoptosis ranking, $Z_{i}$	$\left(\alpha \cdot \text{strength}+\beta \cdot \text{differentiation}+\gamma \cdot \psi \right)/\left(\alpha +\beta +\gamma \right)$

*Note:* ψ denotes the age factor between 0 and 1. λ is the sparsity cost. $a_{m}$ is the average hidden activation and $a_{t}$ the target activation.

Sparse connectivity was implemented via a randomly initialized connectivity mask determining which synaptic weights were allowed to deviate from zero. Although the connectivity of the EC to DG has been estimated to lie at a maximum of ∼2% (Amaral et al. 1990), because our model only has 100 EC neurons, we increased the connectivity to 5%. Young neurons have still comparatively few connections and gain more connections when they grow older. In our simulations, newborn neurons have initially one randomly initialized connection (1% of EC neurons; 1 EC neuron is connected to 5 GCs) with new connections being added and also randomly initialized with increasing age factor.

#### 2.2.2 Neurogenesis

It is estimated that in young rats up to 9000 GCs are being born each day (Cameron and Mckay 2001), although only few of these are thought to survive into maturity (Kempermann et al. 2003). Compared to the pool of mature granule cells neurogenesis thus contributes only a small number of cells (Amaral et al. 2007). To account for that, and unlike other simulations (Aimone et al. 2009) we have a relatively small fraction (15%; see Table 2) of neurons being born throughout the simulation. These cells are initially missing and are born randomly throughout the simulation. This is simulated by resetting their initial age to a randomized low value which effectively shifts the steep area of the Gompertz function in a way that places it within the time window of the simulation. Any new connections are randomly initialized.

#### 2.2.3 Excitability

Young GCs have increased membrane resistance and lower firing thresholds (Liu et al. 1996; Schmidt‐Hieber et al. 2004), making them more excitable than mGCs. This results in higher firing rates, which amplifies learning via STDP. We modulate the leak conductance with age $g_{\text{leak}}=\frac{3}{2}-\frac{1}{2}f\left(t\right)$ (nS), causing higher responsiveness of the membrane potential and consequently higher firing rates.

#### 2.2.4 Apoptosis

It is not entirely clear under which circumstances GCs undergo apoptosis. While only a fraction of cells fully mature in the first place, the death of mature cells seems to be related to their age as well as their activity (Cecchi et al. 2001; Hutchins and Barger 1998). To decide which neurons undergo apoptosis in our model, we adopted the procedure of Finnegan and Becker (2015): all hidden neurons are ranked based on their *age*, mean absolute connection strengths (*strength*), and the standard deviation of their connection strengths (*differentiation*; see Finnegan and Becker 2015 for the motivation) in a 15:25:60 relative importance ratio. At any time, the lowest 10% of neurons in this ranking are considered as apoptosis candidates and their age is reset to 0 if their maturity exceeds $f\left(t\right)\geq 0.95$. If the age of a neuron is reset, all its weights are set to zero. In case the neuron is reborn, its weights get randomly paired with EC neurons and the connections get added over time by re‐initializing the connections randomly and allowing them to deviate from zero.

### 2.3 Training Data and Outcome Measures

The outcome measures of the simulation were memory interference as evident from cued pattern reconstruction in the visible neurons, as well as pattern separation in the neural code in the hidden neurons. We continuously assessed *retroactive* and *proactive* interference throughout the training procedure. To assess memory interference, a dataset was created similar to Finnegan and Becker (2015), consisting of binary patterns with about 20% of the visible neurons being clamped *active*. To cover a wide range of input–output pattern overlap, like Finnegan and Becker (2015), we generated five *seed* patterns, which were then used to generate 10 *prototypes* of each of them by exchanging the activation state of 20% of the active neurons with randomly picked inactive neurons. Ten additional variations were generated from each prototype by similarly resetting another 5% of the active neurons of the prototype. To maximize the spread of possible pattern overlap, the initial seed patterns were chosen to cover as wide of a range of pattern distances between their combinations as possible.

The total data set comprised 10*45 patterns for the 10 groups, plus 10*5 test patterns for the assessment of each proactive and retroactive interference, plus the same amount of neutral phases to assess pattern reconstruction. This resulted in a total of 650 phases.

The composition of the final training data was intended to present a challenge for the model in terms of memory interference by creating different pattern groups with similar but not exactly the same input patterns which had to be learned successively. Each group consisted of nine variations, derived from one prototype of each of the five seed patterns, resulting in 45 patterns per group. The first pattern group contained variations of the first prototype of each seed pattern, the second group variations of the second prototype of each seed pattern, and so on. The variations were sampled randomly from the pool of possible variations after a testing set was created. This serves as the basis for a continual learning task that can be thought to represent ongoing learning in an adapting environment. More details of the data generation can be taken from Table 4.

**TABLE 4:** Parameters for data generation.

Parameter	Value
Length pattern	100
Number of seed patterns	5
Active bits seed pattern	20
Minimum//maximum percent overlap between seed patterns	25%//70%
Number of prototype patterns	50 (10 per seed)
Bits flipped in prototype generation	10
Number of final variations	500 (10 per prototype)
Bits flipped in final variation generation	5
Training patterns per prototype	10
Test patterns per prototype	1

Reconstruction accuracy was assessed by cueing the model with the test stimulus for the duration of one stimulus period, followed by a period with no input during which the model had time to settle into a learned global activation state (see Figure 2). To assess if the resulting global state corresponded to the test pattern, spike counts of the visible (input) neurons and hidden neurons (granule cells) were recorded. We used cosine similarity between the vector of spike counts and the input pattern to assess the accuracy of the reconstruction.

**FIGURE 2:**
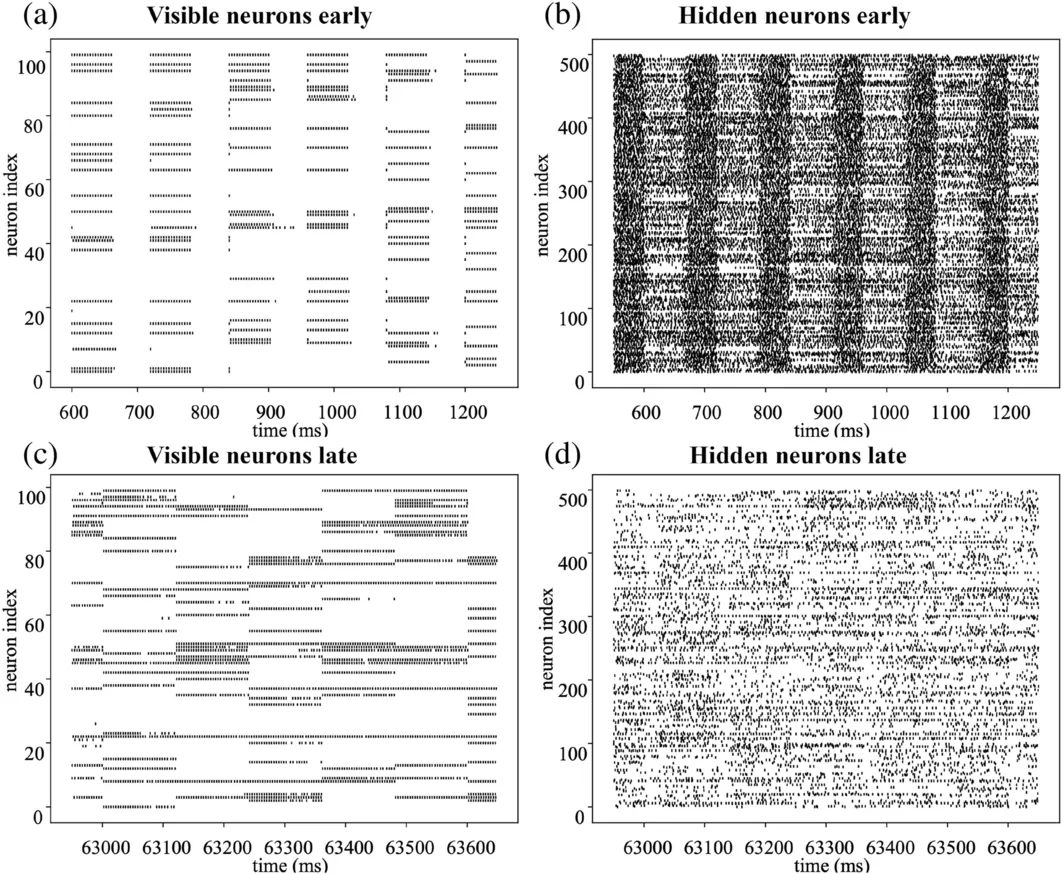
Spiking activity of visible (a and c) and hidden (b and d) neurons early (a and b) and late (c and d) during training. Depicted are ca. 5 time steps. In a, the data‐driven and model driven phases of each time step are clearly differentiable as active and inactive phases. The inactive periods visible in are not the periods where pattern reconstruction is assessed, although the dynamics are similar. (a) Only during active stimulus presentation, clear firing is visible. (b) During learning, hidden neurons produce mostly random spikes. (c) Network retains the primed configuration. (d) Hidden neurons retaining primed configuration.

Proactive interference was measured by testing the pattern reconstruction ability of the model after learning one of the pattern groups by using one randomly selected test pattern corresponding to the prototypes of that group. If previously learned patterns interfered with the learning of new ones, we expect low values for the reconstruction accuracy. Strong proactive interference would be characterized by a reduction in reconstruction accuracy (negative slope) over time. Retroactive interference, on the other hand, was measured by testing pattern reconstruction accuracy after all pattern groups were learned. If newly learned patterns overwrite old patterns, we would expect higher reconstruction accuracies for more recently learned groups than for the first groups (positive slope).

Pattern separation was assessed by computing the absolute deviation:

(8) $$d_{\text{absolute}}=\sum_{j=1}^{N_{h}}|x_{j}-y_{j}|$$

where $x_{j}$ and $y_{j}$ are the re‐scaled spike‐count vectors of two input or hidden patterns respectively. Distances were calculated of every possible combination of input pairs in the pool of patterns consisting of the final pattern group and the final test patterns. These distances can then be related to the distances of their corresponding hidden activations. Input values are rounded to be either 0 or 1, while hidden values were left to be continuous since a partly firing node can also contribute to the encoding. The input distances were then plotted against the distances of their corresponding hidden representations. The resulting figure gives an idea of how the model balances pattern separation and integration (see Yassa and Stark 2011 for an idealized example of such a curve).

### 2.4 Setup

The model was built using Python 3 and the Brian2 package (Stimberg et al. 2019). The code^1^ is based on the one shared by Neftci et al. (2014), although translated from Python 2 to Python 3 and Brian to Brian2. Our specific model had 100 visible and 500 hidden neurons, roughly corresponding to the ratio of EC neurons to GCs (O'Reilly and McClelland 1994).

One run of the model simulated the learning of a changing stimulus over time, creating a continuous learning problem (e.g., see Draelos et al. 2017; Wang et al. 2024). This mimics a changing environment or a task that requires the sequential learning of new, similar components. Throughout the task, learning continues on different, slightly changing patterns, or even an absence of input. Reconstruction performance of any learned pattern group is assessed before a new group is learned (proactive interference measurement; blue in Figure 3). Since a decrease of reconstruction accuracy is expected if previously learned patterns interfere with the learning of later ones, this setup allows for an assessment of proactive memory interference. The final assessment, after all pattern groups have been learned, serves as an assessment of retroactive interference (retroactive interference measurement; dark orange in Figure 3). If accuracy is smaller for earlier groups compared to later groups, the later groups must have interfered with the representation of earlier ones.

**FIGURE 3:**
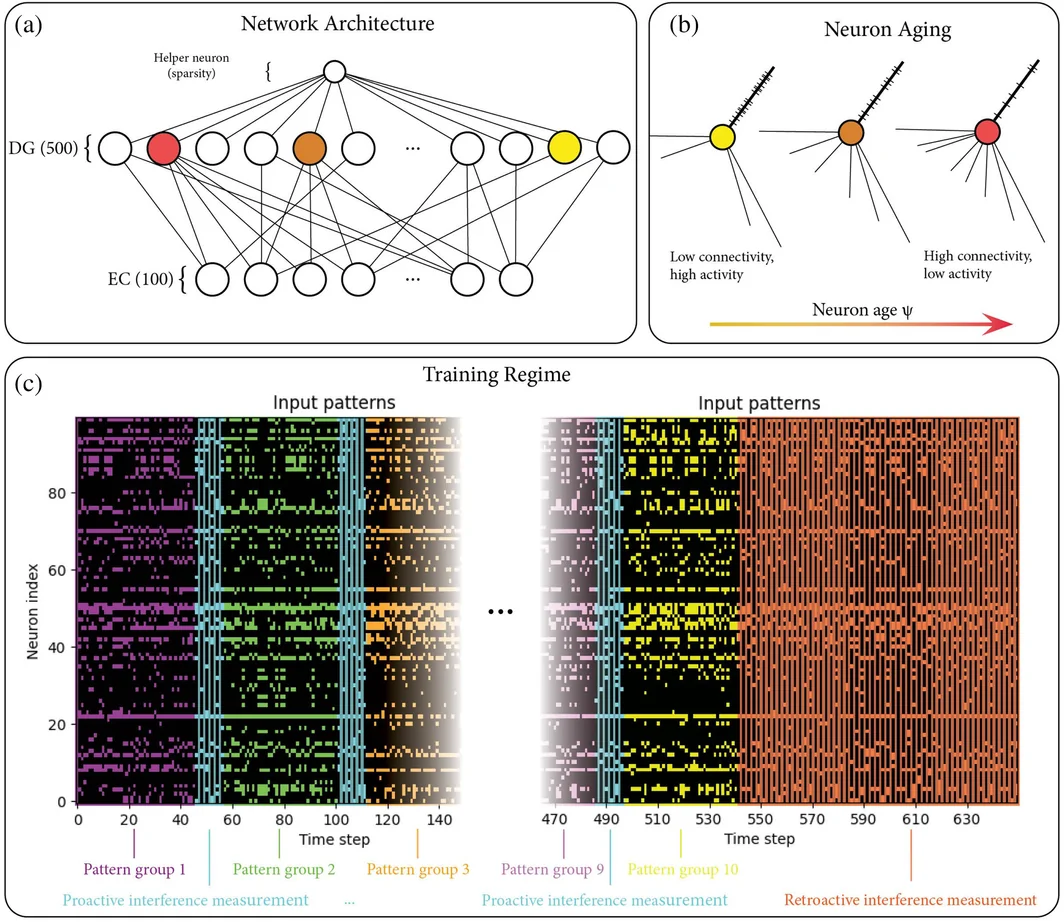
Visualization of architecture, Neuron aging and learning procedure. (a) Architecture of model with 500 neurons in the hidden layer representing granule cells (GC) and 100 cells in the visible layer representing the input from the entorhinal cortex (EC). (b) Neuron aging by getting more connected and less active with age. (c) Visualization of the continual learning procedure. During one simulation run, the model consecutively learns pattern groups one to 10, which represent a slightly changing and noisy environment. After each pattern group, proactive interference is measured by assessing reproduction accuracy of the previously learned pattern group (blue). At the end, retroactive interference is measured by measuring reproduction accuracy of all previously learned pattern groups (dark orange).

Several versions of the model were implemented, in which the various aspects that characterize the maturing of GCs discussed above were manipulated; namely, *aging*, *connectivity level*, the imposition of sparse firing, *hyper‐excitability* of immature GCs, and *apoptosis*. The different model versions are listed in Table 5. In the Base version of the model, the original event‐driven CD learning rule was used, neurons in the visible layer were connected to 5% of the hidden layer neurons, no aging of neurons was implemented, and hence no excitability or apoptosis. This model is compared to a model with neurogenesis (Model 2 in Table 5); to one with neurogenesis and hyper‐excitable imGCs (Model 3); and to one with neurogenesis and apoptosis (Model 4).

**TABLE 5:** Condition description.

Nr.	Model	Aging	Connectivity	Sparse firing	Hp.‐excitability	Apoptosis
1	Base	No	5%	No	No	No
2	Neurogenesis	Yes	1%−5%	No	No	No
3	NG + HE imGCs	Yes	1%−5%	No	imGCs	No
4	NG + Apoptosis	Yes	1%−5%	No	No	Yes
5	Sparse firing	No	5%	All	No	No
6	Ng(mGCs sp. fir) + imGCs sp. con	Yes	1%−5%	mGCs	No	No
7	Ng(mGCs sp. fir) + imGCs f. con	No	5%	mGCs	No	No
8	Ng(mGCs + imGCs sp. fir)	Yes	1%−5%	All	No	No
9	Ng(mGCs sp. fir) + hp.‐exc. imGCs	Yes	1%−5%	mGCs	imGCs	No
10	Ng(mGCs sp. fir) + apoptosis	Yes	1%−5%	mGCs	No	Yes
11	All	Yes	1%−5%	mGCs	imGCs	Yes

Second, the Base model is compared to models with the sparse firing constraint (i.e., the adapted *event‐driven* CD learning rule in Equation 7) applied to all cells (Model 5), and to a model with neurogenesis and only mGCs affected by the sparse firing constraint while the imGCs are sparsely connected (Model 6).

Third, the effect of sparse connectivity in Model 6 is assessed by comparing it to a version in which the imGCs are fully connected at birth (Model 7). Furthermore, the effect of non‐sparse firing in imGCs is compared to a version of Model 6 in which all neurons were subjected to the sparse firing constraint (Model 8).

Finally, the effects of hyper‐excitability of the imGCs and apoptosis on Model 6 are compared in a model with hyper‐excitability but without apoptosis (Model 9), a model without hyper‐excitability with apoptosis (Model 10), and a model with both hyper‐excitability and apoptosis (Model 11).

Each of the models was trained 10 times with different randomization seeds and the resulting data combined for further analysis. Each of these 10 training runs contained 10 groups of patterns that were learned, and for each group a measurement for proactive interference (directly after learning the group) as well as a measure of retroactive interference (in the end of training the model) was recorded. This yielded n=50 similarity measurements per group in both the proactive and retroactive interference measurement which were averaged.

To measure pattern separation, pairs of input patterns and their pattern distance were compared to their respective pairs of output patterns and their pattern distance. The combinations of all possible pairs of input patterns do not result in a uniform distribution of pattern distances. That is, there were more combinations of input patterns that were moderately similar than combinations that were very similar or very different. As a result, intermediate distances provide more precise estimates than distances that are farther away from the mean.

## 3 Results

Figures 4, 5, 6, 7 visualize pro‐active and retro‐active interference of the different model versions. Figures 4, 5, 6, 7 visualize their pattern separation properties. Compared to the base model (model 1 in Table 5), the presence or absence of various characteristics of neural aging markedly changed outcomes. The focus of the presented results lies in isolating the effects of sparse firing as well as sparse connectivity as driving forces of the positive effect of neurogenesis. All results are visualized (from left to right for each of the figures) in terms of their reconstruction accuracy over time (proactive interference), their reconstruction accuracy after learning (retroactive interference), and in terms of their pattern separation dynamics. It is important to note that the scales in the plot describing pattern separation dynamics are not comparable. This is due to the fact that the input vector is binary and the hidden vector continuous between 0 and 1, which causes disproportionately high values for the visible nodes, despite its lower number of neurons.

**FIGURE 4:**
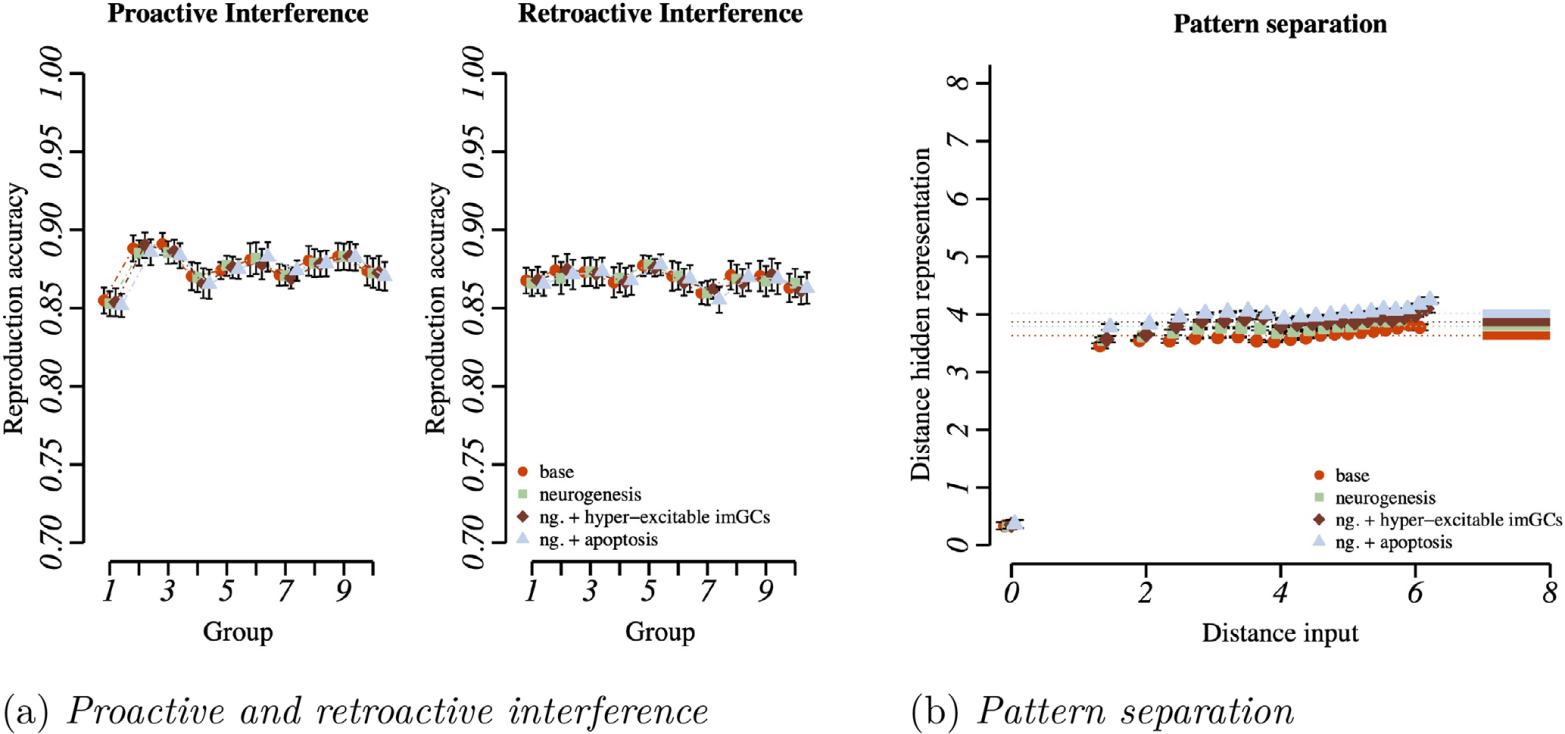
(a) Comparison of proactive and retroactive interference as well as pattern separation for the base model, a model incorporating only neurogenesis, one with neurogenesis and increased excitability and one with neurogenesis and the implementation of neural turnover. There does not seem to be a difference with respect to proactive or retroactive interference. (b) While neurogenesis alone somewhat increases pattern separation, the addition of hyper‐excitability of imGCs seems to increase pattern separation again while neural turnover together with neurogenesis increases pattern separation even more. Bars on the right indicate mean value excluding *y* where *x* = 0.

Figure 4a, depicts the reconstruction accuracy across pattern groups for proactive and retroactive interference measurements. Recall that proactive interference was assessed right after a new pattern was learned, while retroactive interference was assessed after all 10 patterns had been learned. The patterns are marked on the x‐axis in the order in which they were presented to the network. The left panel shows proactive interference as measured by reconstruction accuracy for each of the pattern groups. The base model (red; model 1 in Table 5), the reconstruction accuracy stabilizes after an initial increase, likely due to all pattern groups contained similar patterns: an initial time span is needed to learn the general pattern, during which a steady increase in accuracy can be seen. The fact that no improvement occurs after the second group hints towards a ceiling effect in encoding ability and/or/an equilibrium of proactive interference and encoding ability which prevents a further increase in reconstruction accuracy. With regard to retroactive interference (right panel Figure 4a), the base model shows a steady reconstruction accuracy. Both panels additionally show a neurogenesis model (mint green; Model 2 in Table 5), a neurogenesis + hyper‐excitable imGCs model (brown; Model 3), and a neurogenesis + apoptosis model (light‐blue line; Model 4). None of the models show substantial differences from one another on proactive or retroactive interference.

Figure 4b shows pattern separation for these models. Each of them tends to separate input patterns uniformly; that is, no matter how similar or dissimilar two input patterns are, the patterns of hidden activations all differ roughly to the same degree from one another. There are small but notable differences between models; however, the base model tends to create hidden representations with higher overlap (lower pattern separation) than the neurogenesis model, which in turn separated less than the hyper‐excitable imGCs model. The overall highest pattern separation degree is shown by the neurogenesis + apoptosis model.

Figure 5a compares the proactive and retroactive interference in the base model (red), to a model with all GCs subject to the sparse firing constraint (orange; model 5) and a model with neurogenesis and sparsely firing mGCs and sparsely connected imGCs (turquoise; model 6). The model with sparsely firing GCs (no neurogenesis) performs overall substantially better at reconstruction accuracy (except for the first group). However, clear interference effects emerge: Reconstruction accuracy decreases for pattern groups learned later in the sequence in the proactive interference measurement (left panel), and reconstruction accuracy is lower for earlier learned pattern groups than for later learned pattern groups in the retroactive measurement (right panel). Model 6 incorporates neurogenesis (15% imGCs born throughout the simulation; turquoise line), limits the sparse firing to mGCs, and has imGCs sparsely connected. The effect of these factors is that interference effects visible for model 5 (orange) were mostly mitigated: The drop in accuracy is not present anymore in model 6 during the proactive interference assessments. Similarly, there does not seem to be any retroactive interference in model 6 while the overall reconstruction accuracy improved.

**FIGURE 5:**
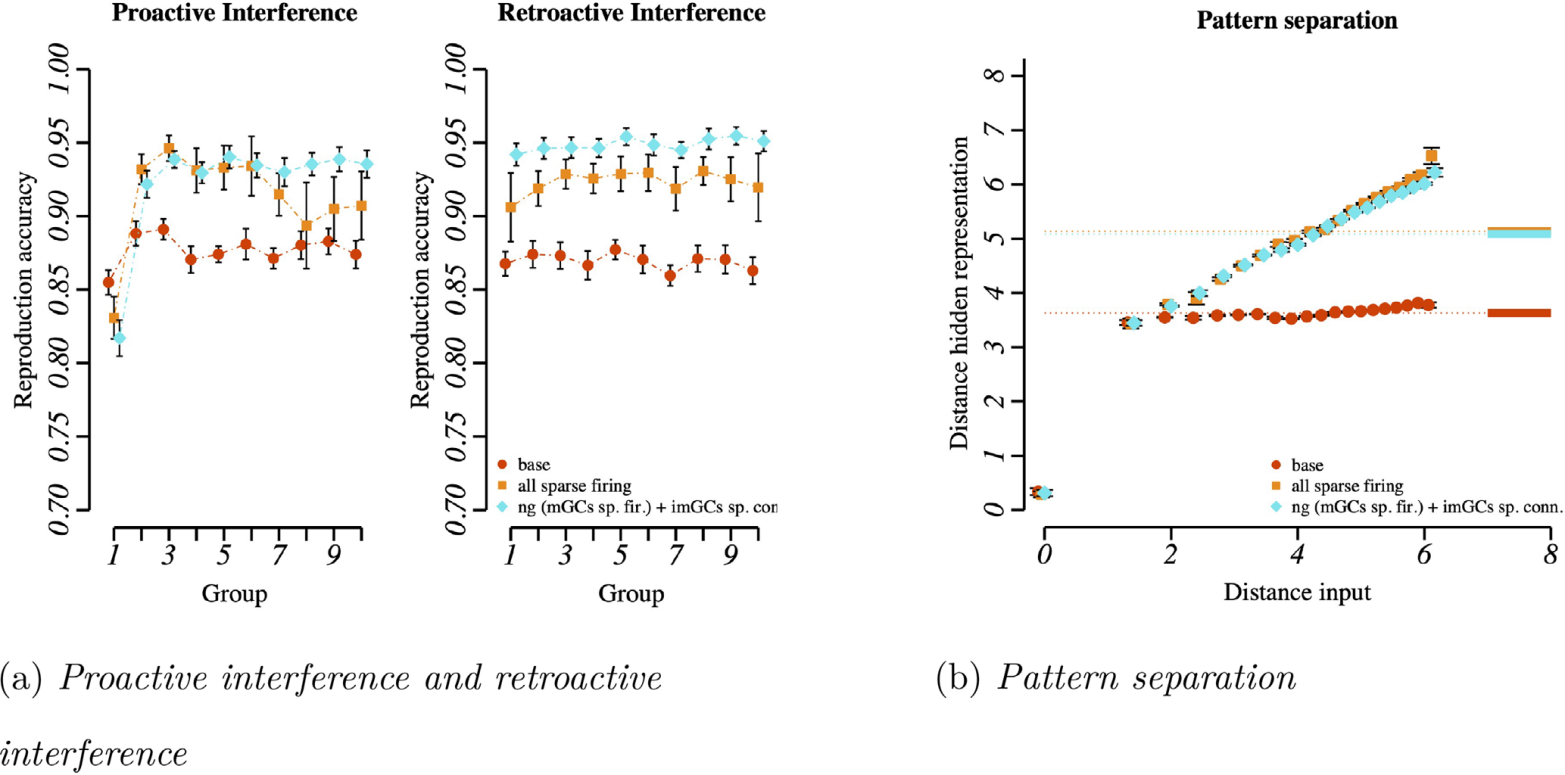
(a) Sparse firing alone (orange) leads to lower proactive and retroactive interference compared to the base model (red). The incorporation of neurogenesis with sparsely connected imGCs and sparse firing limited to mGCs (turquoise) further improves proactive and retroactive interference in terms of reproduction accuracy. (b) Sparse firing alone (orange) changes the pattern separation dynamics compared to the base model (red). The additional implementation of neural aging characteristics (sparsely firing mGCs and sparsely connected imGCS, turquoise) does not seem to improve pattern separation, despite its effect on proactive and retroactive interference. Bars on the right indicate mean excluding *y* where *x* = 0.

These effects correspond to a clear effect in the pattern separation measurements for these models (Figure 5b). While the base model (red) shows uniform pattern separation, the model 5 (orange) shows progressively increasing pattern separation: Similar input patterns somewhat more dissimilar hidden representations, and as input patterns become more dissimilar the hidden representations become increasingly dissimilar. The inclusion of neurogenesis and sparsity constraints does not appear to affect the amount of pattern separation (model 6, turquoise).

To examine the effects of sparse coding constraint and sparse connectivity constraint in more detail, in Figure 6a we compare model 6 with a model with neurogenesis and sparsely firing mGCs but not sparsely connected imGCs (dark green, model 7; in this model, imGCs are immediately as connected as mGCs), and a model that includes neurogenesis and in which both imGCs and mGCs were subjected to sparsely connected and sparsely firing imGCs (light purple, model 8). Both these models show strong proactive and retroactive interference effects as witnessed by the reduction in reconstruction accuracy for later pattern groups in the left panel, and the strongly reduced reconstruction accuracy for earlier pattern groups in the right panel. Interestingly, initially the reconstruction accuracy for model 8 (light purple) seems to be somewhat higher than model 6 (turquoise).

**FIGURE 6:**
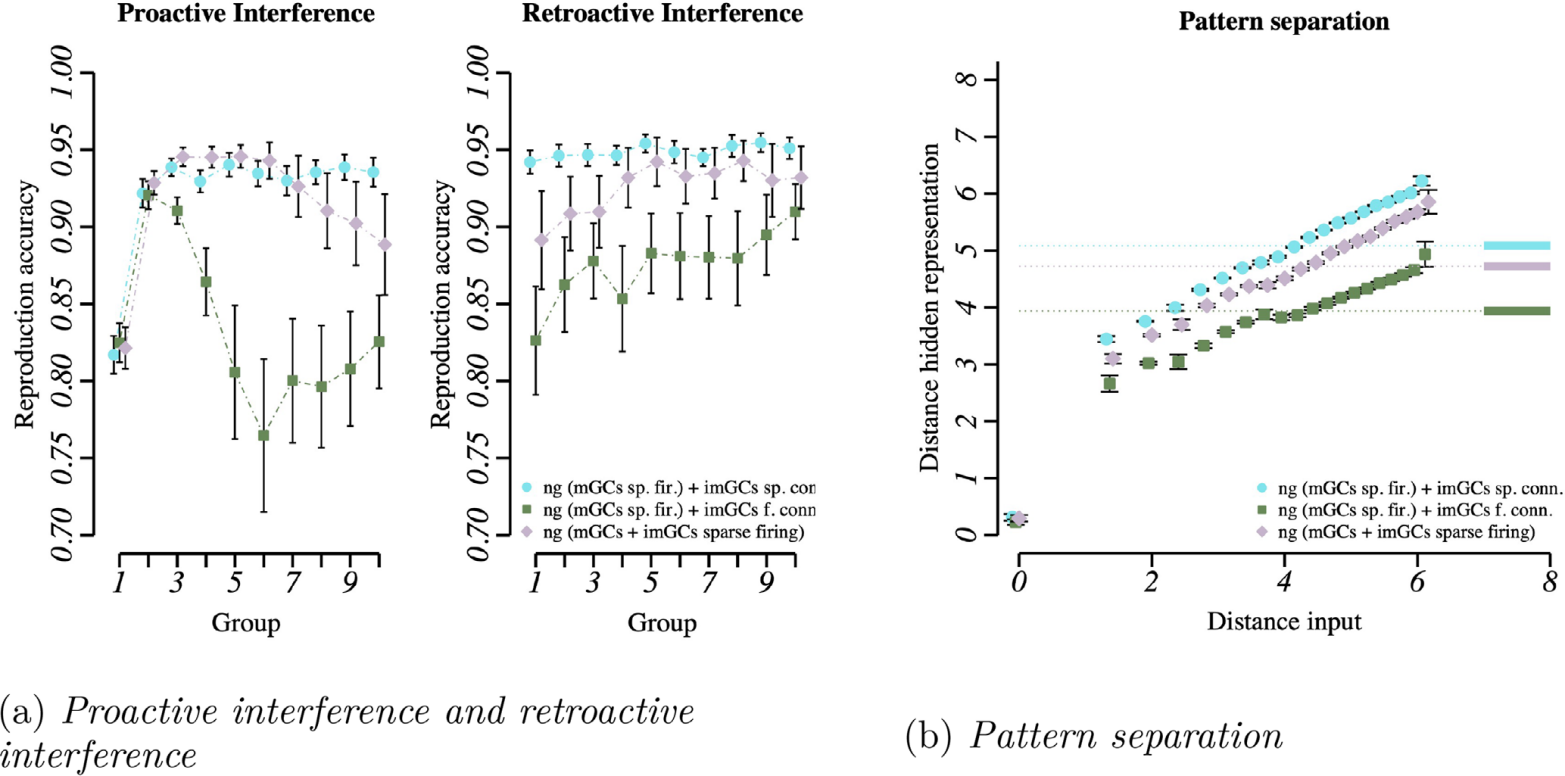
(a) Deviating from the dynamics as found in biological studies such as by allowing imGCs to be as connected as mGCs (dark green) or applying sparse firing to not only mGCs but also imGCs (light purple) strongly impairs learning via proactive and retroactive interference. (b) The effects visible in terms of proactive and retroactive interference are mirrored in their respective pattern separation dynamics, with better performing models having higher overall pattern separation.

These effects are also clearly reflected in the pattern separation degree shown on the right in (Figure 6b). All models show the same progressively increasing pattern separation, although model 7 with neurogenesis and immature granule cells being fully connected (dark green) separates overall much less than model 6 (light blue) and model 8 (light purple).

Figure 7a assesses the effects of hyper excitability and apoptosis by comparing model 6 (turquoise) with a model that in addition to the characteristics of the turquoise one has hyper‐excitable imGCs via increased leak (gray; model 9), a model that in addition to the turquoise one has apoptosis implemented (blue; model 10), and a model that extends the turquoise one with hyper‐excitable imGCs and apoptosis (yellow, model 11). None of the characteristics that go beyond sparse firing and sparse connectivity constraints seem to impact the reconstruction accuracy of the models in any meaningful way, although the model implementing turnover (blue) seems to suffer from higher proactive interference.

**FIGURE 7:**
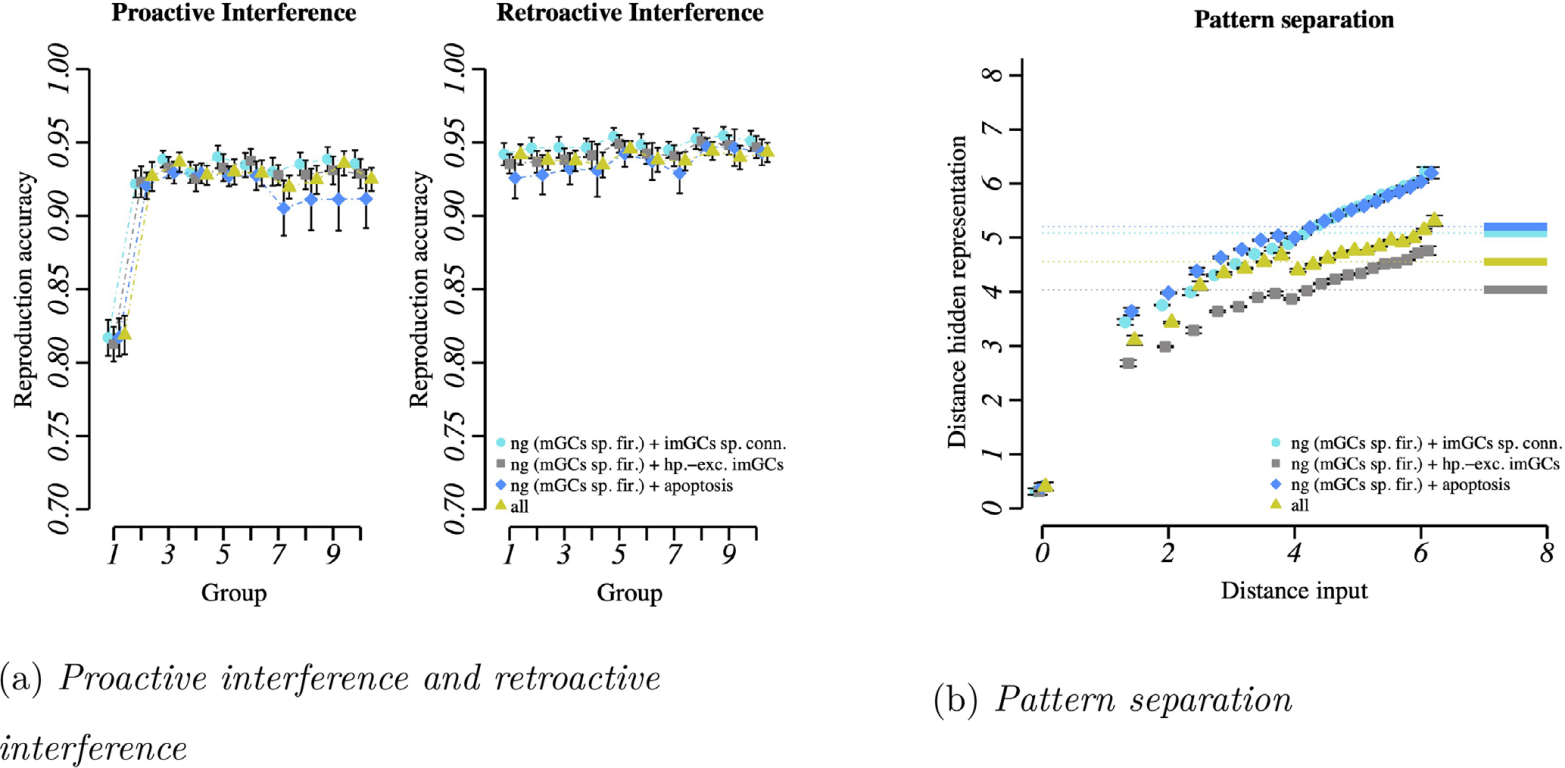
Comparison sparse firing/connectivity model (turquoise) vs. models with added apoptosis and/or hyper‐excitable imGCs. (a) Compared to the sparse firing/sparse connectivity model, the implementation of all neurogenesis characteristics (yellow), results in similar performances in terms of proactive and retroactive interference. The model with added apoptosis (blue) performs somewhat worse than the other models. (b) Pattern separation in the models with apoptosis and/or hyper‐excitability either perform similar or worse than the model with sparse firing and sparse connectivity. Interestingly the model with hyper‐excitable imGCs (gray) shows much lower pattern separation, despite similar interference performance compared to model with sparse firing and sparse connectivity (turquoise). Bars on the right indicate mean value excluding values with zero input distance.

In terms of pattern separation (Figure 7b), however, the models differ quite a bit. Generally, hyper‐excitability (gray), apoptosis (blue), and the combination of both (yellow) seemed to have a detrimental effect on pattern separation degree, with hyper‐excitability alone lowering overall pattern separation strongly compared to all other conditions. Both models involving apoptosis (yellow and blue) seem to flatten the slope of the pattern separation dynamics to some degree compared to model 6 (turquoise). Additionally, the models involving apoptosis show a sudden bump in pattern separation degree, which is, however, likely due to characteristics of the training data and also somewhat visible in other models.

## 4 Discussion

With our simulations, we aimed to investigate whether adult neurogenesis (ANG) can be a mechanism of the putative role of pattern separation of the dentate gyrus (DG). The main focus of the simulation was on the incorporation of ANG including the dynamics that functionally distinguish immature granule cells (imGCs) from mature granule cells (mGCs). Specifically, imGCs were modeled as more excitable, less sparsely firing, and more sparsely connected than mGCs. Additionally, we implemented apoptosis to simulate a steady rate of neural turnover. The implementation of these characteristics was done by adapting the learning rule, connectivity, and neuron dynamics of an sRBM representing an EC input to a layer of GCs.

The results of our simulations were partially consistent with previous studies and expanded on the specific insights on the interplay of the various factors that constitute ANG. Firstly, similar to Finnegan and Becker (2015) we found a considerable improvement with regard to memory interference when implementing ANG and dynamics of aging GCs in our model. Unlike Finnegan and Becker (2015) however, we observed an increase in overall pattern separation degree, consistent with the prevailing hypothesis originating from biological research (Leal and Yassa 2018; Marr 1971; Treves and Rolls 1994; Yassa and Stark 2011). In addition, we found a distinctive shift from uniform to monotonically increasing pattern separation dynamics. Contrary to Finnegan and Becker (2015), who report a decrease in pattern separation degree as a result of neurogenesis dynamics, we did not find such an effect.

One of the main findings of the current study concerns the importance of the sparse firing constraint for mGCs as implemented in our adjusted *event‐driven* CD learning rule (Equation 7). In our simulation, this constraint had the biggest influence on the pattern separation of the network and showed to be necessary for all other aspects of neurogenesis to have an effect. Importantly, as soon as sparse firing was introduced, pattern separation shifted from being constant to being a monotonically increasing. In addition, memory performance improved considerably, at the cost of higher susceptibility to proactive interference and some retroactive interference.

The addition of initially sparsely connected imGCs throughout the simulation almost completely mitigated the interference effects while showing generally higher reconstruction accuracy for testing of old memories. This effect, however, did not coincide with any further increase in pattern separation degree. The precise mechanics of these effects are not entirely clear. Since no increase in pattern separation degree was observed, this improvement in reconstruction accuracy and the reduction of memory interference might be the result of a reduction of overfitting and a more distributed (though not more orthogonal) encoding of stimuli via the continuous availability of new, unspecialized cells.

Importantly, not imposing sparse connectivity in imGCs drastically increased memory interference. The interference was even stronger than when not incorporating neurogenesis at all. This was accompanied by a clear drop in pattern separation, as to be expected. This suggests that sparse connectivity of imGCs is necessary for ANG to be beneficial. The simulations also indicate that sparse firing needs to be limited to mGCs, with pattern separation dropping substantially and retroactive interference increasing when imGCs are subject to this constraint.

Our simulations did not show any meaningful changes in memory performance with the inclusion of hyper‐excitability in imGCs or apoptosis, although pattern separation was affected to some degree. Interestingly, while interference was not affected in the presence of apoptosis, hyper‐excitability, or both, overall pattern separation degree was generally reduced. This suggests that hyper‐excitability tends to reduce pattern separation, which might explain why Finnegan et al. (2017) and others did not observe an increase in pattern separation with their ANG implementation—consistent with findings from Kim and Lim (2024). Further, the observation that pattern separation can decrease without affecting reconstruction accuracy implies that the relationship between the degree of pattern separation and reconstruction accuracy is not a direct one.

While previous studies often found a decrease of pattern separation related to ANG (Aimone et al. 2009; Finnegan et al. 2017; Kim and Lim 2024), we found an overall *increase* in pattern separation for most models incorporating neurogenesis. Importantly, this was only observed in models incorporating sparse firing in mGCs, and not in the unmodified base model. Reasons for these differences might be rooted in our more sparsely connected model (max 5% connectivity) and in the adapted spiking model, which enabled a more realistic implementation of neuron dynamics.

Comparing directly with the research done by Aimone et al. (2009), it is not entirely clear why the findings differ. One possible difference is that they added substantially more imGCs to their model, ultimately more than doubling the number of GCs which makes comparison to our model difficult. Importantly, they also differentiate between pattern integration processes occurring over short timescales compared to pattern separation occurring on longer timescales (Aimone et al. 2009; Kesner et al. 2014; Rangel et al. 2014). Given that our model did not differentiate between long and short time‐scales, a direct comparison is difficult. Other differences with previous research (e.g., Faghihi and Moustafa 2016; Kim and Lim 2024) can also be attributed to the lack of learning and the putative attractor dynamics of hippocampal structures (Knierim and Neunuebel 2016; Neunuebel and Knierim 2014; Wills et al. 2005).

Overall, the present model differs from prior work along two dimensions worth making explicit. First, whereas prior spiking neurogenesis models (Aimone et al. 2009; Faghihi and Moustafa 2016; Kim and Lim 2024) used feedforward architectures that measure pattern separation at encoding only, Aimone et al. (2009) explicitly restricted their analysis to the encoding phase and did not model retrieval, the RBM framework used here is auto‐associative: stored patterns can be reconstructed from partial cues, and proactive and retroactive interference can be measured directly as reconstruction accuracy. Finnegan and Becker (2015) and Finnegan et al. (2017) introduced this attractor framework to the neurogenesis literature but used rate‐coded, non‐spiking neurons. The present model is therefore the first to combine biophysically realistic spiking dynamics with the auto‐associative RBM framework. This combination allows for the simultaneous observation of pattern separation, memory interference and the sparse firing constraint's effect on both. Second, the sRBM architecture generates ongoing activity between stimulus presentations via learned per‐neuron bias inputs and membrane noise, a dynamical regime absent from purely stimulus‐driven models. As noted above, this may contribute to memory performance through spontaneous attractor reactivation, though the model 5 vs. model 6 comparison suggests the structural neurogenesis constraints are the primary driver.

Despite these differences, our findings remain compelling, especially in light of existing neurobiological evidence. Firstly, the dynamics according to which our sparsely firing model carries out pattern separation align with the dynamics described by earlier studies on pattern separation in the DG (Yassa and Stark 2011). As expected, given the ratio of EC to DG cells, our model exhibits the characteristics of a pattern separation stage. This is evident in the models with sparse firing and sparse connectivity, which show increasingly strong separation in the hidden representation given more different input patterns.

Given the isolation of sparse firing as the driver of increased pattern separation and reduced memory interference in our findings, future empirical work could focus on disrupting feedback inhibition mechanisms that drive sparse firing in the DG (some work on this has already been done, with results in line with our simulations, see Morales et al. 2021, who showed that silencing somatostatin‐expressing interneurons and thus reducing feedback inhibition reduces pattern separation).

Overall, our results suggest that the growth of imGCs during learning might modulate the tradeoff between pattern separation and integration, which has long been hypothesized in empirical neuro‐biological studies (Clelland et al. 2009; Creer et al. 2010; Déry et al. 2013; Leal and Yassa 2018; Yassa and Stark 2011). Specifically, our component‐wise findings echo the empirical maturation profile of dentate granule cells. Mature GCs must fire sparsely, and immature GCs must form only a handful of synapses, two constraints that are well documented in vivo. The sparse firing in mGCs (and the unconstrained learning in imGCs) may substantially increase the role that the more easily activated imGCs play in the encoding of novel inputs. At the same time, their sparse connectivity could serve as a mechanism to limit their role in the encoding of stimuli that are already encoded.

Somewhat surprisingly, the inclusion of hyper‐excitability in imGCs did not have any additional beneficial effects. However, the increased learning from more frequent firing could have a similar effect as imGCs unconstrained in their firing rates. Still, the sparse firing of mGCs already caused a relative difference in firing rate between imGCs and mGCs, which aligns with the proposition that mostly the imGCs are the active population of cells and responsible for a large part of the memory encoding (Alme et al. 2010).

One limitation of our design is that the current model is a compromise between biological plausibility and the intended comparability between empirical and simulation studies. While the hippocampus itself acts as a short‐term memory storage, the dentate gyrus itself has been described as a pattern separation stage only. For the sake of interpretability, our model describes broader physiological dynamics than just the functioning of the dentate gyrus. Future research should extend this foundation to represent a larger part of, or even the entire trisynaptic circuit. This would strengthen the analogy between the RBM and the hippocampal network as it involves more than just a single layer of reciprocal connection as in the simple RBM (Finnegan et al. 2017). Further, the effect of different cell types and their specific dynamics can be modeled more precisely in the future. With more realistic neuron dynamics to model GCs such as proposed in Chavlis et al. (2016), the inclusion of mossy fibers and the explicit and cell‐type‐resolved modeling of feedback inhibition (Temprana et al. 2015), a simulation could benefit from considerably higher anatomical accuracy. Another point that future studies could focus on is the absolute size of the model, as ours may have suffered from its relatively small size. A larger model could implement a more realistic ratio of connectivity between EC and DG neurons, reducing issues related to overfitting as a result of the large number of hidden nodes compared to the visible ones. Finally, upcoming research should extend the continual learning paradigm to allow for pattern separation and integration measures to be taken for short as well as long timescales, allowing for more direct comparisons to previous research such as the model by Aimone et al. (2009).

## 5 Conclusion

By embedding adult neurogenesis into a biophysically grounded, STDP‐trained spiking restricted Boltzmann machine, we demonstrate that the combination of sparse newborn‐to‐mature connectivity and activity‐dependent sparse firing is sufficient to boost pattern separation while simultaneously protecting stored memories from interference. Our analyses show that sparse firing of mature granule cells, rather than transient hyper‐excitability alone, is the primary driver of this improvement, offering a mechanistic explanation for why earlier non‐spiking RBM studies reached the opposite conclusion. Our model remains deliberately minimalist: it omits the recurrent CA3 loop, restricts STDP to pair‐based timing, and operates at a scale well below the full dentate gyrus. Nevertheless, the principles uncovered here—age‐dependent sparsification of synaptic targets and activity budgets—provide clear, testable predictions for future in vivo and in vitro studies, such as reduced receptive‐field overlap among mature granule cells after neurogenic bursts.

## Funding

The authors have nothing to report.

## Data Availability

The data that support the findings of this study are openly available in spikingdentategyrus at https://github.com/van‐der‐meer/spikingdentategyrus.

## Endnotes

^1^
The code can be accessed via: https://github.com/danvdm/spikingdentategyrus.
